# Examining the Theoretical Framework of Behavioral Activation for Major Depressive Disorder: Smartphone-Based Ecological Momentary Assessment Study

**DOI:** 10.2196/32007

**Published:** 2021-12-06

**Authors:** Claire Rosalie van Genugten, Josien Schuurmans, Adriaan W Hoogendoorn, Ricardo Araya, Gerhard Andersson, Rosa Baños, Cristina Botella, Arlinda Cerga Pashoja, Roman Cieslak, David Daniel Ebert, Azucena García-Palacios, Jean-Baptiste Hazo, Rocío Herrero, Jérôme Holtzmann, Lise Kemmeren, Annet Kleiboer, Tobias Krieger, Ewelina Smoktunowicz, Ingrid Titzler, Naira Topooco, Antoine Urech, Johannes H Smit, Heleen Riper

**Affiliations:** 1 Department of Research and Innovation GGZ inGeest Specialized Mental Health Care Amsterdam Netherlands; 2 Department of Psychiatry Amsterdam Public Health Institute Amsterdam University Medical Center, Vrije Universiteit Amsterdam Netherlands; 3 Department of Clinical, Neuro and Developmental Psychology Vrije Universiteit Amsterdam Netherlands; 4 Institute of Psychiatry Psychology and Neurosciences King’s College London London United Kingdom; 5 Department of Behavioural Sciences and Learning Linköping University Linköping Sweden; 6 Centre for Psychiatry Research Department of Clinical Neuroscience Karolinska Institutet Stockholm Sweden; 7 Polibienestar Research Institute University of Valencia Valencia Spain; 8 Institute of Health Carlos III CIBERObn CB06 03/0052 Madrid Spain; 9 Department of Personality, Evaluation and Psychological Treatment Faculty of Psychology University of Valencia Valencia Spain; 10 Department of Basic and Clinical Psychology and Psychobiology Faculty of Health Sciences Jaume I University Castellon de la Plana Spain; 11 Department of Population Health London School of Hygiene & Tropical Medicine London United Kingdom; 12 Faculty of Psychology SWPS University of Social Sciences and Humanities Warsaw Poland; 13 Lyda Hill Institute for Human Resilience Colorado Springs Colorado Springs, CO United States; 14 Department for Sport and Health Sciences Technical University Munich Munich Germany; 15 Eceve, Unit 1123 Inserm University of Paris Paris France; 16 Health Economics Research Unit Assistance Publique-Hôpitaux de Paris Paris France; 17 Mood Disorders and Emotional Pathologies Unit Pôle de Psychiatrie, Neurologie et Rééducation Neurologique University Hospital Grenoble Alpes Grenoble France; 18 Department of Clinical Psychology University of Bern Bern Switzerland; 19 Department of Clinical Psychology and Psychotherapy Institute of Psychology Friedrich-Alexander-University Erlangen-Nürnberg Erlangen Germany; 20 Centre for m2health Palo Alto University Palo Alto, CA United States; 21 Institute of Telepsychiatry University of Southern Denmark Odense Denmark; 22 Faculty of Medicine University of Turku Turku Finland

**Keywords:** depression, behavioral activation, theoretical framework, ecological momentary assessment, random-intercept cross-lagged panel model, behavior, framework, EMA, smartphone, mental health, treatment, engagement, mood

## Abstract

**Background:**

Behavioral activation (BA), either as a stand-alone treatment or as part of cognitive behavioral therapy, has been shown to be effective for treating depression. The theoretical underpinnings of BA derive from Lewinsohn et al’s theory of depression. The central premise of BA is that having patients engage in more pleasant activities leads to them experiencing more pleasure and elevates their mood, which, in turn, leads to further (behavioral) activation. However, there is a dearth of empirical evidence about the theoretical framework of BA.

**Objective:**

This study aims to examine the assumed (temporal) associations of the 3 constructs in the theoretical framework of BA.

**Methods:**

Data were collected as part of the “European Comparative Effectiveness Research on Internet-based Depression Treatment versus treatment-as-usual” trial among patients who were randomly assigned to receive blended cognitive behavioral therapy (bCBT). As part of bCBT, patients completed weekly assessments of their level of engagement in pleasant activities, the pleasure they experienced as a result of these activities, and their mood over the course of the treatment using a smartphone-based ecological momentary assessment (EMA) application. Longitudinal cross-lagged and cross-sectional associations of 240 patients were examined using random intercept cross-lagged panel models.

**Results:**

The analyses did not reveal any statistically significant cross-lagged coefficients (all *P*>.05). Statistically significant cross-sectional positive associations between activities, pleasure, and mood levels were identified. Moreover, the levels of engagement in activities, pleasure, and mood slightly increased over the duration of the treatment. In addition, mood seemed to carry over, over time, while both levels of engagement in activities and pleasurable experiences did not.

**Conclusions:**

The results were partially in accordance with the theoretical framework of BA, insofar as the analyses revealed cross-sectional relationships between levels of engagement in activities, pleasurable experiences deriving from these activities, and enhanced mood. However, given that no statistically significant temporal relationships were revealed, no conclusions could be drawn about potential causality. A shorter measurement interval (eg, daily rather than weekly EMA reports) might be more attuned to detecting potential underlying temporal pathways. Future research should use an EMA methodology to further investigate temporal associations, based on theory and how treatments are presented to patients.

**Trial Registration:**

ClinicalTrials.gov, NCT02542891, https://clinicaltrials.gov/ct2/show/NCT02542891; German Clinical Trials Register, DRKS00006866, https://tinyurl.com/ybja3xz7; Netherlands Trials Register, NTR4962, https://www.trialregister.nl/trial/4838; ClinicalTrials.Gov, NCT02389660, https://clinicaltrials.gov/ct2/show/NCT02389660; ClinicalTrials.gov, NCT02361684, https://clinicaltrials.gov/ct2/show/NCT02361684; ClinicalTrials.gov, NCT02449447, https://clinicaltrials.gov/ct2/show/NCT02449447; ClinicalTrials.gov, NCT02410616, https://clinicaltrials.gov/ct2/show/NCT02410616; ISRCTN registry, ISRCTN12388725, https://www.isrctn.com/ISRCTN12388725

## Introduction

Most psychotherapeutic treatments for depression are underpinned by a clear theoretical framework of how a specific therapy is supposed to engender change in patients’ mood states. In light of the fact that various psychotherapies have been proven to be effective for treating depression (eg, [1-4]), one would perhaps assume that theoretical frameworks are also evidence based. However, demonstrating that a therapy is effective is not the same as providing evidence or explanations as to how it actually works [5-8], that is, the process through which a certain variable leads to change in another variable [6,7]. In addition to effectiveness studies that target an overall treatment package, understanding how therapy targets the interplay between specific factors that are believed to be of importance can ultimately give rise to specific treatment recommendations or improvements to treatment protocols (eg, [6-10]). Conducting a verification of the underlying theoretical framework of a form of psychotherapy can help optimize treatment strategies (eg, [6-10]), that is, direct better, stronger, or different treatment strategies that underpin the critical processes of the treatment (eg, [6-10]).

**Figure 1:**
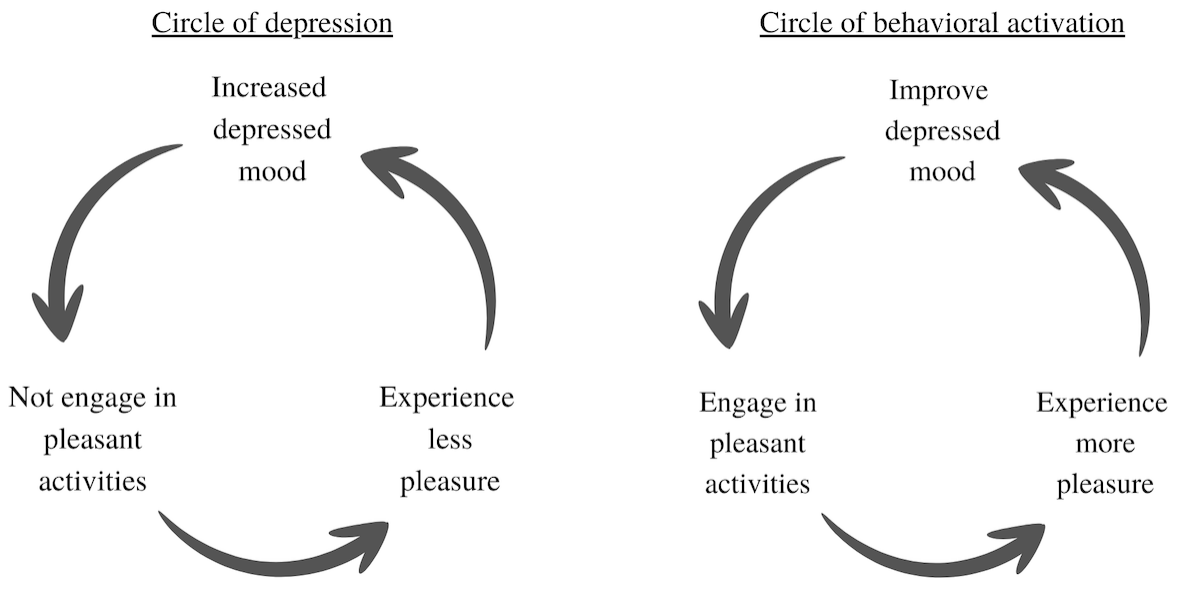
Circles of depression and BA based on the theoretical framework of depression by Lewinsohn et al [14,15]. BA: behavioral activation.

One such effective psychotherapeutic intervention for depression is behavioral activation (BA). The empirical evidence for BA is both extensive and convincing; it can be offered either as a stand-alone treatment or within the setting of cognitive behavioral therapy (CBT) [11-13]. BA is based on Lewinsohn et al’s [14,15] theory of depression, which purports that when a person is depressed, they tend to engage less in pleasant or meaningful activities, resulting in them experiencing less pleasure, which, in turn, leads to an increased depressed mood, and so on. From this perspective, persons end up in a vicious circle of depression [14,15], as depicted in the left panel of Figure 1.

Through BA, patients learn from self-monitoring their everyday activities and related mood that the way they behave affects the way they feel, while conversely, the way they feel affects the way they behave [11,12,16]. Therapists motivate their patients to schedule and engage in everyday pleasant activities [12,17]. Patients are informed that engaging in these activities may not lead them to immediately experience greater pleasure and that experiencing pleasure may not enhance their mood straightaway. Rather, they are told that this is a process that requires time and that positive results will follow in due course as they continue to engage in pleasurable activities [12,17]. The circle of BA, as it is referred to in Lewinsohn et al’s [14,15] model is shown in the right panel of Figure 1. As one can see in Figure 1, BA aims to break the vicious circle of depression by increasing the patient’s engagement in pleasant activities.

Currently, there is a relative dearth of empirical evidence available concerning the assumed causal pathways in the theoretical framework of BA [10,18-20]. Janssen et al [20] sought to address this gap in extant knowledge by conducting a systematic review in which they investigated the mediators of BA for depression. In this review [20], the evidence for the mediating role played by both engaging in pleasant activities and experiencing pleasure was not consistently replicated across the considered studies. However, the authors did conclude that their review was not without its limitations. First, some of the included studies were of poor methodological quality, while different questionnaires were used to assess the mediators. Moreover, the results of the mediational analysis did not necessarily explain the processes via which change occurs, insofar as most studies solely focused on unidirectional relationships, in addition to lacking temporal dependency [9,20]. It is thought that the reciprocal interactions and longitudinal associations between different variables determine psychological functioning [21]. Thus, to successfully delineate the theoretical framework of BA, further research is needed that considers reciprocal interactions and longitudinal associations by using ecological momentary assessment (EMA) methods [8,10,20].

EMA methods allow us to trace temporal pathways of change across different variables among patients during the course of treatment [9,10,20,22]. With EMA methods, or real-time monitoring, persons are routinely asked to report on their mood and other related phenomena while they are in their own ecological habitat (eg, [23-25]). Traditionally, EMA was conducted via paper-and-pencil diaries as well as via stand-alone technical devices [25,26]. Today, EMA is often facilitated by smartphone-based applications [25,27,28]. The fact that phenomena are measured close to their occurrence helps to avoid recall bias as much as possible [29,30]. This fact is especially pertinent with respect to patients suffering from mood disorders, as prior research has shown that recall among this patient group is confounded by current feelings, not to mention greater recall of unpleasant activities than pleasant activities [31-33]. The past several decades have seen a surge in the use of EMA methods in mood disorder research.

This paper aims to examine the assumed (temporal) associations of the 3 constructs in the theoretical framework of BA among major depressive disorder (MDD) patients, who monitored their engagement in pleasant activities, the pleasure they experienced as a result of conducting these activities, and their related mood states using smartphone-based EMA during the course of blended CBT (bCBT) in routine mental health care (MHC). bCBT integrates face-to-face (f-t-f) sessions with both web- and smartphone-based components into 1 treatment protocol [34]. Data from a subsample of the patients receiving bCBT and smartphone-based EMA (N=240) as part of the European Comparative Effectiveness Research on Internet-based Depression Treatment treatment-as-usual (TAU) (E-COMPARED) trial were used to investigate the longitudinal reciprocal associations [35,36].

## Methods

### Participants, Procedure, and Treatment Protocol

E-COMPARED is a randomized controlled, non-inferiority trial that was conducted across 8 European countries. The principal aim of the trial was to compare both the clinical effectiveness and cost-effectiveness of bCBT to treatment-as-usual (TAU) for MDD. For the trial, a generic bCBT protocol was established [35,36]. The study protocol of E-COMPARED gives a complete overview of the bCBT protocol [36]. In short, the core components of the bCBT protocol mirrored traditional f-t-f CBT: (1) psychoeducation, (2) BA, (3) cognitive restructuring, and (4) relapse prevention. However, with bCBT, f-t-f sessions were replaced by and alternated with online modules [34,37]. The themes of the f-t-f sessions matched the content of the online sessions. The more practical components were delivered online (ie, psychoeducation, completing homework assignments). The focus of the health care professional during the f-t-f session was on process-related treatment outcomes (ie, discussion of feelings and thoughts), discussing homework assignments, and providing support [36]. Although the protocols included the same core components (including BA), bCBT was delivered in the primary care of specialized MHC and therapists were granted some freedom to customize the protocol to their local needs. To make sure the protocol suited their local context, the therapists were allowed to adapt the treatment duration and the ratio between the number of f-t-f sessions and online sessions. In addition, therapists were allowed to include some additional components (eg, mindfulness or problem solving), but this could not take up more than 25% of the total treatment (f-t-f and online sessions combined). Moreover, based on local availability, different web- and smartphone-based applications were used. Table 1 shows the bCBT format per country.

**Table 1:** Blended cognitive behavioral therapy format per country.

Country	Type of care	Treatment duration (weeks)	Face-to-face sessions, n	Web-based modules, n	Total number of sessions, n	Sequencing
Germany	Primary	11-13	6	10	16	Alternate
Poland	Primary	6-10	7	6	13	Alternate
England	Primary	11	5	6	11	Alternate
Spain	Primary	10	10	10	20	Alternate
Sweden	Primary	10	4	6	10	Alternate
The Netherlands	Specialized	18-20	10	9	19	Alternate
France	Specialized	16-20	8	8	16	Alternate
Switzerland	Specialized	18	9	9	18	Alternate

As with traditional f-t-f CBT, the BA component in this study was based on Lewinsohn et al’s [14,15] theory of depression and aimed at increasing patients’ engagement in activities. The BA component started early in treatment and remained a recurring topic in the (f-t-f) sessions throughout the course of treatment. During the f-t-f sessions, the patient was motivated by the health care professional to schedule and engage in (potential) pleasant activities. The patient could (re)read the rationale of BA online, read a so-called activity-list that could be used for inspiration, and use the platform as a tool to specify and schedule which activities to engage in that week. A smartphone-based EMA application was used to monitor engagement in activities, pleasure experienced as a result of these activities, and the mood state over the course of treatment. Although the f-t-f and online sessions were only approximately (alternated) once a week, the patient was encouraged to take an active role in their therapy and to practice in their own environment in between the sessions.

Between February 2015 and December 31, 2017, patients were recruited from primary care (Germany, Poland, Spain, Sweden, and the United Kingdom) and outpatient departments of specialized MHC settings (France, the Netherlands, and Switzerland) [35,36]. Patients were asked by their health care professionals whether they were willing to take part in the study. The inclusion criteria were as follows: (1) must be at least 18 years of age; (2) meet the *Diagnostic and Statistical Manual of Mental Disorders*, *Fourth Edition* (DSM-IV), criteria for MDD, as confirmed by Mini International Neuropsychiatric Interview (M.I.N.I.) version 5.0 [38,39]; and (3) report mild-to-severe depressive symptoms (score of ≥5) on the Patient Health Questionnaire-9 (PHQ-9) [40,41]. The exclusion criteria were as follows: (1) already receiving psychological treatment for depression in a primary or specialized MHC setting; (2) be at high risk for suicide or have a DSM-IV diagnosis of substance dependence, bipolar disorder, psychotic illness, or obsessive compulsive disorder, as confirmed by M.I.N.I. version 5.0 [38,39]; (3) not able to comprehend the spoken and written language of their country of residence; (4) not have access to a computer with a fast internet connection; and (5) not have a smartphone compatible with the Android operating system or be unwilling to carry a smartphone provided by the research team. For more in-depth information about the specifics of both country and setting recruitment procedures, please see elsewhere [35,36].

Patients who met the inclusion criteria (N=943) were randomly allocated to receive either bCBT (n=476) or TAU (n=467). For the purposes of this study, only patients who were randomized to receive bCBT were initially selected, since patients who were allocated to the TAU group were not invited to complete the smartphone-based EMA measures. Of the 476 bCBT patients, 152 did not receive treatment (never attended the first f-t-f session, dropped out after the first f-t-f session, never logged onto the platform) or did not provide any weekly EMA reports. Of the remaining 324 patients, all patients reported on their mood, but 84 of them failed to complete reports on their levels of activities or pleasure. This led to an analytic sample of 240 patients who reported on all 3 variables of interest (activities, pleasure, and mood) in the weekly EMA reports. Potential selection bias was examined by analyzing potential differences in terms of demographic and clinical characteristics between the E-COMPARED patients who were randomly allocated to receive bCBT but did not meet the study’s inclusion criteria (n = 476 – 240 = 226) and patients who were included in this study (N=240). The results of these analyses are presented in the Result section.

### Measures

#### Demographic and Clinical Characteristics

At baseline, information about demographic and clinical characteristics was gathered. The basic demographics included age, gender, and educational level. This information was obtained through a web-based questionnaire. The clinical characteristics included current MDD diagnosis and other (comorbid) psychiatric diagnoses, severity of depression, and use of antidepressant medication. The presence of current MDD and current comorbid psychiatric disorders (dysthymia, panic disorder with or without agoraphobia, agoraphobia, social phobia, generalized anxiety disorder, posttraumatic stress disorder) was defined according to DSM-IV criteria [39] and established using M.I.N.I. version 5.0 [38]. Patients reported on the severity of their depression by completing a web-based version of the PHQ-9 [40-42]. This questionnaire contains 9 items, each of which covers 1 DSM-IV criterion of MDD [39]. Questions are answered on a scale from 0 (not at all) to 3 (nearly every day), as experienced during the prior 2-week period. Sum scores indicate both the presence and the severity of depressive symptoms: none (0-4), mild (5-9), moderate (10-14), moderately severe (15-19), and severe depressive (20-27) symptoms [40,41]. The last item of the PHQ-9 evaluates suicidal ideation (ie, passive thoughts of death or self-injury). The researchers of E-COMPARED did not actively monitor or respond to reports of suicidal ideation, as this was not a stand-alone online treatment service. The MHC professional providing the therapy was considered well qualified and trained to identify and address suicide risk in their patients.

### Smartphone-Based EMA of Pleasant Activities, Pleasure, and Mood

Information about activities, that is, the level of engagement in pleasant activities, on the day of reporting was gathered through the following question: “To what extent did you accomplish pleasant activities today?” The question was answered on a visual analogue scale (VAS) that ranged from 1 to 10, with 1 precision digit after the decimal point and higher scores indicating more engagement in pleasant activities. Pleasure, that is, the subjective appraisal of the pleasure experienced through these activities, on the day of reporting was measured through the question “How much did you enjoy activities today?” This question was answered on a VAS scale that ranged from 1 to 10, with 1 precision digit after the decimal point and higher scores indicating greater pleasure experienced. Lastly, information pertaining to the patients’ current mood was collected through the following question: “How is your mood right now?” This question was answered on a VAS scale that ranged from 1 (worst) to 10 (best), with 1 precision digital after the decimal point. Higher scores thus indicated more engagement in pleasant activities, greater pleasure experienced, and better mood on the day of reporting.

The EMA protocol varied over the course of the treatment. During both the first and the last 7 days of treatment, patients were prompted to rate their mood on 3 separate occasions each day (around 10:00 AM, 8:00 PM, and a random time between 10:00 AM and 10:00 PM). During these specific weeks, at the 8:00 PM prompt, patients were also invited to report on their level of pleasant activities and experienced pleasure. From the second week until the last week of treatment, patients were prompted to rate their mood once a day at a random time between 10:00 AM and 10:00 PM. Moreover, on 1 random evening (8:00 PM) each week, patients were also invited to rate their engagement in pleasant activities, experienced pleasure, and mood. Although patients were instructed to complete the questions as quickly as possible, they were given a time frame of 60 min. Additionally, patients were also free to report their mood at any time other than the fixed prompts. The EMA protocol varied over the course of treatment, as the EMA component was used for supportive means in the treatment. The first week was for patients to get used to the EMA application, but it was considered unrealistic to expect from patients in a clinical setting to complete a full diary every day throughout the course of treatment [35,36].

For this study, weekly averages of the EMA reports were calculated in order to ensure that all patients had an equal number of measurement points. The weekly averages of the 3 questions were calculated over a 3-month period, which resulted in 12 weekly EMA reports of activity, pleasure, and mood for each patient, as 3 months was deemed an appropriate timespan for examining the process of change, given the average length of bCBT protocols [36].

### Statistical Analysis

Descriptive statistics were calculated for both baseline demographics and clinical characteristics. To answer the main research questions, linear mixed model (LMM) and random-intercept cross-lagged panel model (RI-CLPM) analyses were conducted. First, we performed multiple imputation (MI, m=100) to impute the missing EMA weekly reports. In the data set, 18%, 54%, and 54% of the 12-week reports were missing for mood, pleasure, and activity, respectively. Full information maximum likelihood (FIML) and MI are 2 types of techniques that are considered best for handling missing data [43,44]. When conducting an RI-CLPM in RStudio, the default setting to handle missing observations is FIML [45]. We, however, chose to apply MI since this technique allows for a more convenient way to incorporate auxiliary variables in the model when running an RI-CLPM in RStudio. Auxiliary variables are additional covariates that are included in the model next to the variables ultimately analyzed in the final analysis. It is argued that adding auxiliary variables can substantially improve the handling of missing data [46].

We performed MI using the Amelia II-R-package (version 1.7.6) [47] as this allows for MI of time series data. The package uses a bootstrap-based expectation-maximization bootstrapping algorithm to impute missing observations. It uses all of the information present in the data set, allows for previously known information to be incorporated into the imputation model, and provides diagnostics of the model [47]. In this study, the patient ID was included as a fixed effect. Time was considered by including leads (previous measurement [t–1]) and lags (next measurement [t+1]) into the imputation model. Previously known information was included by setting a logical bound between 1 and 10, as this was the answer range for the EMA questions. Auxiliary variables included gender, age, educational level, comorbid DSM-IV diagnoses at baseline, PHQ-9 at baseline, PHQ-9 after 3 months, and antidepressant usage at baseline. Diagnostics of the imputation model were checked by examining overimputation diagnostic plots. Please see Multimedia Appendix 1 [48-54] for more information about both missing data and the MI procedure.

**Figure 2:**
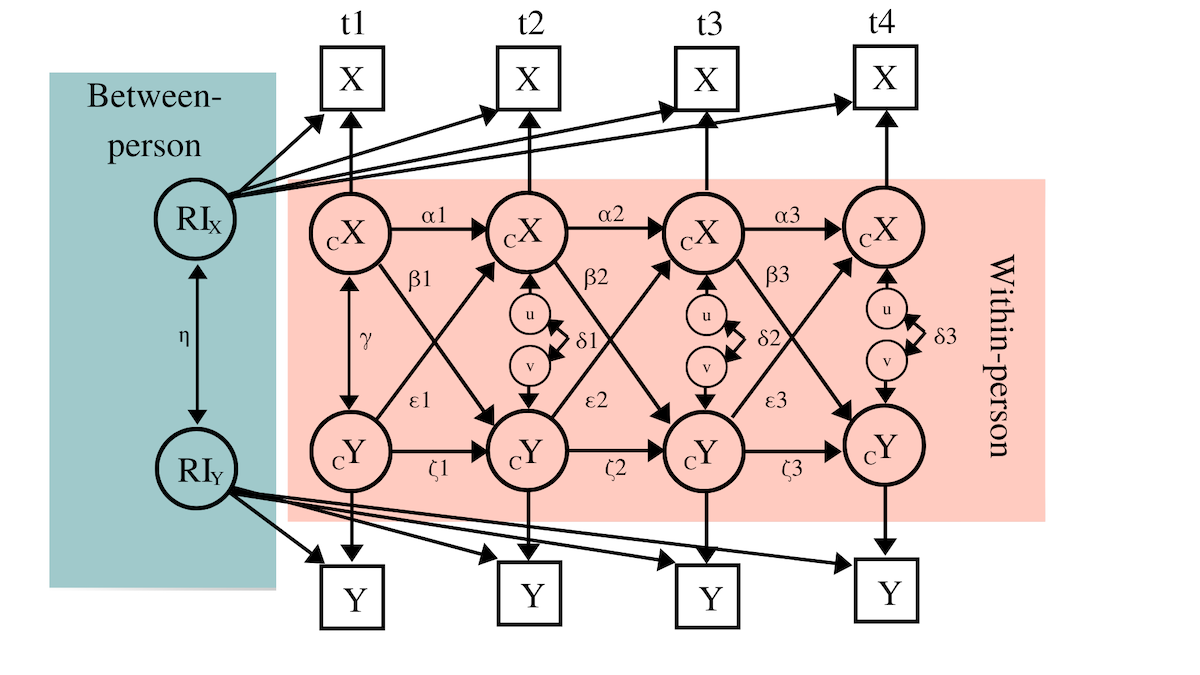
RI-CLPM for 4 measurement points. *α* and *ζ* are autoregressive regression coefficients; *γ* and *δ* are same-week (residual) covariances, *β* and *ε* are cross-lagged regression coefficients, and *η* is between-person correlation. Based on Hamaker, Kuiper, and Grasman [55]. RI: random intercept; RI-CLPM: random-intercept cross-lagged panel model.

Next, the development of engaging in activities, pleasure experienced, and mood state over the duration of the treatment was examined with 3 separate LMMs. Weekly averages of the EMA reports were added as dependent variables. To test our main research questions, unconstrained RI-CLPMs were estimated. Again, the weekly averages of the EMA reports were analyzed. The RI-CLPM, as proposed by Hamaker, Kuiper, and Grasman [55], is an extension of the traditional CLPM [56]. The CLPM is an expedient method for describing cross-lagged associations between variables [56]. However, 1 key drawback of the traditional CLPM is that within-person effects cannot be extracted; this is problematic, given that these effects reflect the intraindividual processes that are needed in order to be able to draw conclusions about how changes over time in one variable are linked to changes over time in another variable, with respect to the same person. The RI-CLPM decomposes the between- and within-person effects. Figure 2 provides a visual representation of an RI-CLPM.

The RI-CLPM involves:

- Autoregression coefficients (*α* and *ζ*), which represent the carry-over effect (eg, X_t_ on X_*t* +1)_. A negative coefficient indicates that if the score for X in one week is above the overall mean, then the score for X in the following week is likely to be below the overall mean, and vice versa. Conversely, a positive coefficient indicates that a higher or lower score for X in one week corresponds to a higher or lower score for X in the following week.
- The cross-lagged coefficients (*β* and *ε*) indicate the extent to which 2 variables influence each other. The coefficients show the extent to which any deviation in variable X in one week is related to any deviation in variable Y in the following week, when controlled for the autoregression of Y.
- The covariance (*γ*) indicates the association between X and Y in the first week, while the same-week residual covariances (*δ*) indicate the covariant change, that is, the extent to which a within-person change in X is associated with a within-person change in Y in the same week.
- The association between the random intercepts (RIs; *η*), which reflects the between-person effects over the course of the treatment.

The model fit of the RI-CLPMs was evaluated with (1) the *P* value of the *χ*^2^ test statistic, (2) the standardized root-mean-square residual (SRMR), (3) the standardized root-mean-square error of approximation (RMSEA) [57], and (4) the comparative fit index (CFI) [58]. For an RI-CPLM to fit the data well, (1) the *χ*^2^ test statistic must be statistically nonsignificant (*P*>.05), (2) the SRMR must be ≤0.08, (3) the RMSEA must be ≤0.05, and (4) the CFI must be ≥0.95 [57-60].

All analyses were performed in RStudio (R version 4.0.2.). The mitml-R-package was used to prepare the MI data sets for pooled analyses [61]. The LMMs were conducted using the nlme-R-package [62]. For the RI-CLPM, we derived our model syntax from the (basic) model syntax provided by Mulder and Hamaker [45]. To run the model syntax on the MI data sets, we needed the semTools-R-package [63]; this package provided the lavaan interface for the MI data sets [63,64]. Rubin’s rules were applied to pool the results across the MI data sets [65]. *P*<.05 was considered statistically significant. A more detailed description of the RI-CLPM [55,66], the model syntax for the RI-CLPM [45], the Amelia II, lavaan, mitml, nmle, and semTools packages [47,61-64,67] and Rubin’s rules [65] can be found elsewhere.

## Results

### Sample Characteristics

Table 2 shows the baseline demographics and clinical characteristics of the analytic sample. The sample comprised 240 participants, 66% (158/240) of which were female. The mean age was 37.3 years (SD 13.2), while 9% (21/240), 34% (80/240), and 58% (139/240) of the patients studied at an elementary, secondary, and higher educational level, respectively. One or more comorbid DSM-IV diagnoses were reported by 59% (142/240) of the patients, while 30% (71/240) were currently using antidepressant medication. Finally, 13% (31/240) of the patients reported mild, 35% (84/240) reported moderate, 30% (72/240) reported moderately severe, and 22% (53/240) reported severe depressive symptoms at baseline.

The analyses that were conducted to examine potential selection bias demonstrated that there was no difference in terms of demographics and clinical characteristics between the E-COMPARED patients who were randomly allocated to receive bCBT but did not meet the study’s inclusion criteria (see the Methods section) (n=476–240=226) and patients who were ultimately included in the study (N=240).

**Table 2:** Baseline demographics and clinical characteristics of the study sample (N=240).

Characteristic		Patients, N (%)
**Gender**		
	Female	158 (66)
	Male	82 (34)
**Educational level**		
	Elementary	21 (9)
	Secondary	80 (33)
	Higher	139 (58)
**Comorbid DSM-IV^a^ diagnoses^b^**		
	0	98 (41)
	1	77 (32)
	2 or more	65 (27)
**Antidepressant use**		
	No	169 (70)
	Yes	71 (30)
**PHQ-9^c^**		
	Mild	31 (13)
	Moderate	84 (35)
	Moderately severe	72 (30)
	Severe	53 (22)

^a^DSM-IV: Diagnostic and Statistical Manual of Mental Disorders, Fourth Edition.

^b^Current comorbid diagnoses included the DSM-IV diagnoses of dysthymia, panic disorder with or without agoraphobia, agoraphobia, social phobia, generalized anxiety disorder, and posttraumatic stress disorder.

^c^PHQ-9: Patient Health Questionnaire-9.

### Development of Engagement in Pleasant Activities, Experiencing Pleasure, and Mood During the Course of the Treatment

Over the course of a 12-week period, patients provided weekly EMA reports concerning their activities, pleasure experienced, and mood. Since missing data were imputed with MI techniques (see Multimedia Appendix I), 12 weekly EMA reports for all 3 variables were available for each patient. On average, engagement in activities (B=0.02, SE 0.01, *t*_849_=2.03, *P*=.04), pleasure experienced (B=0.03, SE 0.01, *t*_943_=2.95, *P*=.003), and mood (B=0.04, SE 0.01, *t*_1350_=6.2, *P*<.001) of patients all increased slightly during the course of the treatment. Age, gender, depression severity, and antidepressant usage were not considered confounders in any of the analyses.

### Longitudinal Reciprocal Associations in the BA Circle

#### Reciprocal Associations Between Engaging in Pleasant Activities and Experiencing Pleasure

**Figure 3:**
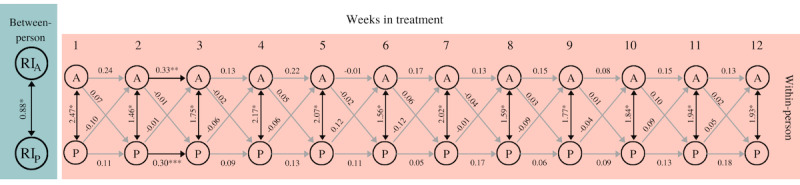
Simplified RI-CLPM engaging in pleasant activities and experiencing pleasure. The between-person double-headed arrow represents a correlation. Within-person double-headed arrows represent (residual) covariances; single-headed arrows display standardized regression coefficients. Light-gray arrows represent nonsignificant covariances/coefficients. *P<.001, **P=.01, ***P=.02. A: activity; P: pleasure; RI: Random Intercept; RI-CLPM: random-intercept cross-lagged panel model.

Figure 3 shows a simplified model for the reciprocal associations between engaging in pleasant activities and experiencing pleasure. The fit indices indicate that the model fits well (*χ^2^*_217_=52.35, *P*=.99, SMSR=0.05, RMSEA=0.00, CFI=1.00).

At the within-person level, none of the cross-lagged coefficients were statistically significant; engagement in activities was not predictive for experiencing pleasure, nor was the opposite the case. Regarding the autoregression coefficients, only 2 autoregressive paths were significant: the autoregressive path of engaging in activities between weeks 2 and 3 (B=0.33, SE 0.12, *P*=.01) and the autoregressive path of experiencing pleasure between weeks 2 and 3 (B=0.30, SE 0.12, *P*=.02). The positive significant autoregressive pathway indicates that if the level of activities engaged in during week 2 was above the overall mean, then the level of activities engaged in during week 3 was also likely to be above the overall mean. This was also the case with respect to experiencing pleasure in weeks 2 and 3. Moreover, both the covariance between activity and pleasure in the first week (covariance=2.47) as well as the residual covariances from weeks 2 to 12 (range residual covariance=1.46-2.07) were statistically significant (all *P*<.001). This means that increased engagement in pleasant activities was associated with an increase in the pleasure experienced in the same week.

At the between-person level, engaging in activities and experiencing pleasure were strongly correlated (*r*=0.88, *P*<.001). This means that over the course of the treatment, patients who engaged more in pleasant activities also reported experiencing greater pleasure than those who engaged less in pleasant activities.

#### Reciprocal Associations Between Experiencing Pleasure and Mood

**Figure 4:**
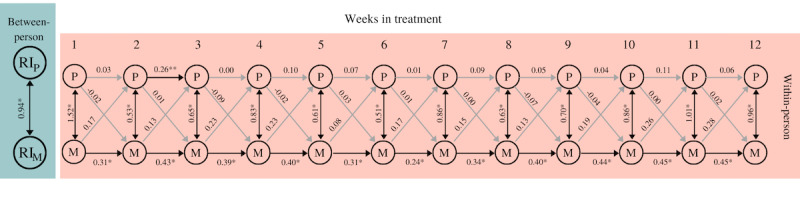
Simplified RI-CLPM experiencing pleasure and mood. The between-person double-headed arrow represents a correlation. Within-person double-headed arrows represent (residual) covariances; single-headed arrows display standardized regression coefficients. Light-gray arrows represent nonsignificant covariances/coefficients. *P<.001, **P=.01. M: mood; P: pleasure; RI: Random Intercept; RI-CLPM: random-intercept cross-lagged panel model.

Figure 4 shows a simplified model for the reciprocal associations between experiencing pleasure and mood. The fit indices indicate that the model fits well (*χ^2^*_217_=91.67, *P*=.99, SRMR=0.08, RMSEA=0.00, CFI=1.00).

At the within-person level, none of the cross-lagged coefficients were statistically significant; the level of pleasure was not predictive for mood 1 week later, nor vice versa. With regard to the autoregression coefficients, the autoregressive path between weeks 2 and 3 was statistically significant with respect to experiencing pleasure (B=0.26, SE 0.10, *P*=.01), while all the coefficients of mood appeared to be positive and significant (B=0.24-0.45, *P*<.001). This positive significant autoregressive pathway thus indicates that if the level of experiencing pleasure (or mood) in week t was above the overall mean, then the level of experiencing pleasure (or mood) in week t+1 was also likely to be above the overall mean. Moreover, both the covariance between experiencing pleasure and mood in the first week (covariance=1.52) as well as the residual covariances from weeks 2 to 12 (range residual covariance=0.51-1.01) were statistically significant (all *P*<.001). This means that an increase in experienced pleasure was associated with an increase in mood in the same week.

At the between-person level, experiencing pleasure and mood were strongly correlated (*r*=0.94, *P*<.001). This means that over the duration of the treatment, patients who experienced more pleasure also reported being in a better mood than patients who experienced less pleasure.

#### Reciprocal Associations Between Mood and Engaging in Pleasant Activities

**Figure 5:**
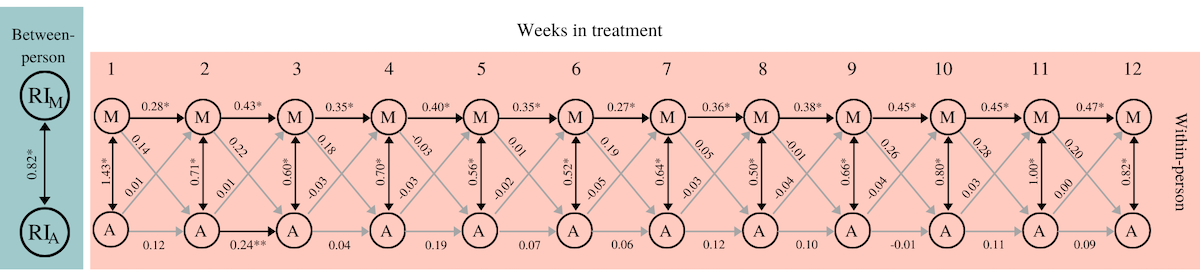
Simplified RI-CLPM mood and engaging in pleasant activities. The between-person double-headed arrow represents a correlation. Within-person double-headed arrows represent (residual) covariances; single-headed arrows display standardized regression coefficients. Light-gray arrows represent nonsignificant covariances/coefficients. *P<.001, **P=.02. A: activity; M: mood; RI: Random Intercept; RI-CLPM: random-intercept cross-lagged panel model.

Figure 5 shows a simplified model for the reciprocal associations between mood and engagement in pleasant activities. The fit indices indicate that the model fits well (*χ^2^*_217_=98.65, *P*=.99, SRMR=0.08, RMSEA=0.00, CFI=1.00).

At the within-person level, none of the cross-lagged coefficients were statistically significant. Mood was not predictive of engagement in activities 1 week later, nor vice versa. With regard to the autoregression coefficients concerned, autoregressive paths for mood were found to be positive and significant (B=0.28-0.46, *P*<.001), while for engagement in activities, only the autoregressive path between weeks 2 and 3 (B=0.24, *P*=.02) was significant. Moreover, both the covariance between mood and engagement in activities in the first week (covariance=1.43) as well as the residual covariances from weeks 2 to 12 (range residual covariance=0.50-1.00) were statistically significant (all *P*<.001). This means that an increase in mood was associated with an increase in engagement in activities in the same week.

At the between-person level, there was a strong correlation between mood and engagement in activities (*r*=0.82, *P*<.001). This means that over the course of the treatment, patients who experienced improved mood also reported higher levels of engagement in activities.

## Discussion

### Principal Findings

This study is a first attempt in examining the (temporal) associations between the 3 constructs of the theoretical framework of BA, which is grounded in Lewinsohn et al’s [14,15] theory of depression, among MDD patients during the course of bCBT. We found no evidence of a temporal relationship between the 3 variables of interest: engagement in pleasant activities, experiencing pleasure, and mood. However, the results did reveal cross-sectional positive relationships between the 3 variables. Moreover, the levels of engagement in activities, pleasure, and mood increased slightly over the course of the treatment. In addition, mood appeared to be self-predictive over time, which was not the case for either engagement in activities or pleasure. Finally, over the course of the treatment, strong positive correlations between engagement in activities, pleasure, and mood were identified at the between-patient level.

### Comparison With Previous Literature

First, we were interested in examining the temporal dimension of the theoretical framework of BA. In this respect, our findings did not reveal any temporal relationships between engagement in activities and the subsequent pleasure and mood experienced in the following weeks. This result was somewhat surprising as it is not in accordance with Lewinsohn et al’s [14,15] theory of depression, which clearly posits a temporal relationship between engagement in activities and pleasure, pleasure and mood, and mood and activities (see Figure 1). This raises the question of whether the lack of temporal relationships in our study stems from the selected lag (weekly interval).

The selected lag was a consequence of the chosen sampling scheme in the treatment protocol. We could only use the data that were available, and the E-COMPARED study was not designed to investigate the theoretical framework of BA. To avoid false-negative or false-positive findings, it is vitally important to choose the right lag; however, this is difficult as the right lag cannot be standardized but, rather, is completely dependent on the research topic [68,69]. In the case of our study, it could well be that the temporal relationships would have been revealed if the time intervals between the subsequent measurements had been shorter (eg, daily rather than weekly EMA reports).

The question of whether the data were suitable for illustrating temporal relationships is further evoked by the fact that same-week relationships between the 3 examined variables were identified [68,69]. That is, in those weeks when a patient (1) engaged in more pleasant activities, they also reported an increase in pleasure; (2) experienced more pleasure, they also reported a better mood; and (3) reported a better mood, they also engaged in more pleasant activities. Although these within-patient associations are potentially in line with the theoretical framework of BA [14,15], since these same-week relationships are cross-sectional, it cannot be established whether there is causal dominance, equal reciprocal relationships, or cyclical relationships.

Moreover, the analyses highlighted between-patient effects; patients who engaged more in pleasant activities over the course of the treatment also reported experiencing more pleasure. Patients who experienced more pleasure also reported a better mood, while patients who reported a better mood also reported engaging in more activities over the course of the treatment. Although we were primarily interested in intrapatient processes and causal dominance cannot be established, the between-patient effects do complement the intrapatient effects, insofar as the positive relationship between the 3 variables of interest at the between-patient level does fit within the scope of the theoretical framework of BA [14,15]. To the best of our knowledge, this was the first (EMA) study to examine these effects over the course of treatment within a sample of adult MDD patients in routine MHC; prior studies have either focused on comparing healthy controls with depressed persons, who are often recruited from the general population (eg, [70-72]), or investigated activities or pleasure as a mediator of pre- to postdepression severity change after depression treatment [20,73].

### Limitations

In addition to the question of whether the selected time lag was appropriate, the results should also be considered with a further limitation in mind, namely the proportion of missing weekly EMA reports. In particular, the adherence rates for the engagement in activity and pleasure reports were relatively poor (both 46%). However, we contend that we were able to mitigate this problem by carrying out MI on the missing weekly EMA reports.

### Clinical Implications and Future Research

This study addresses a noteworthy gap in the extant literature and, as such, can be regarded as constituting an important first step toward establishing evidence for the theoretical framework of BA among MDD patients. To the best of our knowledge, this represents the first study to investigate intrapatient processes of BA during the course of (b)CBT treatment. The results lend at least some empirical support for the underlying theoretical framework of a therapy that is regarded by the American Psychological Association [74] as 1 of the recommended treatments for adult depression. A next step would be to replicate the study across a different data set, possibly with smaller time intervals between the measurements. Moreover, in our study, we focused on whether we could find associations between the 3 key constructs that are part of the theoretical framework of BA for MDD. We do know that BA starts early in treatment and remains a recurring topic in the (f-t-f) sessions throughout the entire course of treatment. However, based on our data, we could not tease apart the influence of other intervention components from the influence of BA, as BA was part of CBT. Whether BA succeeds in activating MDD patients would be an interesting question that should be explored in future work.

### Conclusions

The results of our study are partially in accordance with the theoretical framework of BA. The analyses demonstrated statistically significant cross-sectional relationships between levels of engagement in activity, pleasure experienced as a result of these activities, and mood. However, as we did not reveal any statistically significant temporal relationships, no conclusions could be drawn concerning possible causality. A shorter measurement interval (eg, daily rather than weekly EMA reports) might be more conducive to detecting potential underlying temporal pathways. Consequently, future research should use an EMA methodology to further investigate these temporal associations, based on theory and how the treatments are presented to patients.

## Appendix

Multimedia Appendix 1 Multiple imputation of missing data.

## Abbreviations

BA: behavioral activation
bCBT: blended cognitive behavioral therapy
CFI: comparative fit index
DSM-IV: Diagnostic and Statistical Manual of Mental Disorders, Fourth Edition
E-COMPARED: European Comparative Effectiveness Research on Internet-based Depression Treatment versus treatment-as-usual
EMA: ecological momentary assessment
FIML: full information maximum likelihood
f-t-f: face-to-face
LMM: linear mixed model
MDD: major depressive disorder
MHC: mental health care
MI: multiple imputation
PHQ-9: Patient Health Questionnaire-9
RI: random intercept
RI-CLPM: random-intercept cross-lagged panel model
RMSEA: root-mean-square error of approximation
SRMR: standardized root-mean-square residual
TAU: treatment-as-usual
VAS: visual analogue scale
