# Supplementary material for: Examining the Theoretical Framework of Behavioral Activation for Major Depressive Disorder: Smartphone-Based Ecological Momentary Assessment Study
Source: JMIR Ment Health. 2021 Dec 6;8(12):e32007. doi: 10.2196/32007 (PMC8727050; doi:10.2196/32007)
Supplement: Multimedia Appendix 1 [file mental_v8i12e32007_app1.doc]

## Multimedia Appendix 1: Multiple imputation of missing data.

Missing data are the rule rather than the exception in clinical research [70]. Full maximum likelihood (FIML) and multiple imputation (MI) are two types of techniques that are considered best in handling missing data [43,44]. When conducting a RI-CLPM in R-Studio, the default setting to handle missing observations is FIML [45]. We, however, chose to apply MI since this technique allows for a more convenient way to incorporate auxiliary variables in the model when running a RI-CLPM in R-Studio. Auxiliary variables are additional covariates that are included in the model next to the variables ultimately analyzed in the final analysis. It is argued that adding auxiliary variables can substantially improve the handling of missing data [46].

MI creates *m* copies of the dataset, thus replacing the missing observations in each dataset with independent random draws from the distribution of the missing values [48-50,65]. The final analysis is fitted to each imputed dataset and the results are then pooled using Rubin’s rules [51,65]. One of the advantages of MI is that it can increase the power of the analysis, insofar as all the information present in the dataset can be used [48-50,65]. MI can be used to prepare data for several types of analysis, including structural equation modelling (such as the Random–Intercept Cross–lagged Panel Model RI-CLPM) [52,53].

In this paper, the Amelia II-R-package (version 1.7.6) was used for MI, as this package allows for the MI of time series data [47]. This package is an easy-to-use tool that uses a bootstrap-based Expectation-Maximation Bootstrapping algorithm to impute missing observations. Amelia II uses all information present in the dataset, allows for previously known information to be incorporated into the imputation model, and provides diagnostics of the model [47]. One of the assumptions of Amelia–II is that the missing data are missing at random (MAR). This means that it is assumed that the missingness depends on the observed data (=not missing), but is independent from the unobserved data (=missing) [51,65]. According to Rubin’s terminology [58,71], data can also be missing completely at random (MCAR) or missing not at random (MNAR). MCAR means that the missing data is unrelated to any observed (=not missing) and unobserved (=missing) data, while MNAR means that the missingness depends on the unobserved (=missing) data [51,65]. Over the years, research has been conducted on both if and how one can test whether missing data is MAR, MCAR or MNAR [54]. However, these studies focused on cross-sectional and not time series data. Fortunately, the Amelia II manual states that the MAR assumption can be made more plausible by including additional covariates into the imputation dataset than just those that are ultimately envisioned to be used in the final analysis [47].

Overall, we performed 100 imputations (*m*=100). In the dataset, 18%, 54% and 54% of the weekly reports were missing for mood, pleasure and engagement in activity reports, respectively. Alongside the weekly reports on mood, pleasure and engagement in activity, the imputation model also included auxiliary variables (the covariates gender, age, educational level, comorbid DSM-IV diagnoses at baseline, Patient Health Questionnaire-9 questionnaire (PHQ-9) at baseline, PHQ-9 after 3 months, antidepressant usage at baseline). Patient ID was included as a fixed effect. Time was taken into account by including leads (previous measurement [t-1]) and lags (next measurement [t+1]) into the imputation model. Prior known information was included by setting a logical bound between 1-10, as this was the answer range (visual analogue scale) for the EMA reports. According to the diagnostic plot, the imputation models of activity, pleasure and mood, all fit well.

A full description of the diagnostic plots is beyond the scope of this paper and can be found in the manual of the Amelia II-R-package [47].
